# Zika virus and Assisted Reproductive Technology: to test or not to test, that is the question. Is it an unnecessary cost? The first two months of mandatory testing in an outbreak area in Rio de Janeiro, Brazil

**DOI:** 10.5935/1518-0557.20160038

**Published:** 2016

**Authors:** Maria do Carmo B. de Souza, Veronica Raupp, Fernanda Sobrinho, Mariana Menezes, Tatiana R Panaino, Maria A Tamm, Ana C A Mancebo, Ana L R Costa, Roberto A Antunes

**Affiliations:** 1 Fertipraxis Centro de Reprodução Humana, Rio de Janeiro, RJ, Brazil; 2 Hospital Federal da Lagoa. Rio de Janeiro, RJ, Brazil

**Keywords:** Zika virus, immunology, in vitro fertilization

## Abstract

**Objective:**

Infection by the Zika virus is a Public Health Emergency of International Concern as defined by the World Health Organization. Resolution no. 72, issued by the Collegiate Board of the Brazilian Health Surveillance Agency (ANVISA) on March 30, 2016, made ZKV testing mandatory prior to procedures involving germ cells and tissues. This paper aims to discuss the aforementioned Resolution from the standpoint of evidence and cost-effectiveness of the measures taken within the first two months of mandatory testing.

**Methods:**

The medical staff at the clinic looked into the steps needed to comply with the new rules and checked laboratories in the city to perform the tests with their lead times and costs, health insurance refunds, data maintenance capabilities, how to contact patients, decision-making processes in ongoing cases, deadlines for implementation, in addition to exchanging ideas with other clinics and gathering information from the guidelines being produced. A SWOT analysis was performed.

**Results:**

A total of 152 tests were performed within the first two months of mandatory testing, in five different clinical situations: one previously symptomatic woman with a negative PCR test before starting the cycle; two asymptomatic women had positive IgM (1.3%) and negative PCR tests on days 25 and 60; one husband enrolled as a suspect with a negative RT-PCR on day 13 and another untested suspected case; a couple decided to have their oocytes cryopreserved because the husband's test result was not available on pickup day. The mean cost of USD 200 per couple is equivalent to 1.2 day of the stimulation protocol. The staff worked more efficiently and was able to respond promptly to the increased demand for ZKV testing; however, the tests failed to reassure patients of the safety of the procedure and increased costs.

**Conclusion:**

The testing requirement for asymptomatic patients prior to ART should be reviewed.

## INTRODUCTION

The World Health Organization (WHO) has elevated the disease by the Zika virus (ZKV) to the status of a Public Health Emergency of International Concern (PHEIC). The disease is transmitted primarily via infected Aedes aegypti or Aedes albopictus mosquitoes. Theses vectors feed primarily on humans, and often bite multiple individuals in a single meal; their bite is almost imperceptible, and the mosquitoes live in close association with human habitation (Petersen *et al.*, 2016). The most common symptoms of ZKV disease are fever, rash, joint pain, and conjunctivitis (pink eyes). The infection usually presents with mild symptoms lasting for several days to a week. Most people do not feel sick enough to go to a hospital, and deaths by Zika infection are very rare. For this reason, many people might not realize they have been infected (CDC, 2016). More than 1,500,000 cases were estimated to exist in Brazil alone (Table 1) (Ministry of Health, 2016 a,b); cases of ZKV infection have been reported in other South and Central American nations. There are many guidelines to patients and government actions in progress, but so far early fetal viremia, even during embryo life, has been more closely associated with congenital anomalies (Noronha *et al.*, 2016).

**Table 1:** Infections by Zika virus in Brazilian States confirmed by lab workup. Projections for 2015, Ministry of Health, Brazil, 2016

State	Lower limit	Upper limit
Alagoas	4,023	29,066
Amazonas	3,119	34,264
Bahia	19,216	132,274
Ceara	38,485	77,469
E Santo	6,481	34,190
Maranhao	1,481	60,067
M Grosso	8,202	28,410
Para	6,357	71,400
Paraiba	6,013	34,558
Parana	42,008	97,118
Pernambuco	34,579	81,303
Piaui	3,237	27,875
Rio de Janeiro	15,918	143,985
Rio G de Norte	4,761	29,947
Rondonia	2,911	15,383
Roraima	1,450	4,399
São Paulo	236,494	386,249
Tocantins	8,767	13,182
BRASIL	443,502	1301, 140

* Estimates for lower limits were discarded cases of Dengue and the proportions of cases in the French Polynesia, based on the population of each state. Reflection on the potential dispersion of the virus, more than 80% of asymptomatic cases

The frequency and risk factors for transmission are still unknown, but robust evidence indicates that ZKV can be transmitted from the mother to the fetus during pregnancy; viral antigen and RNA have been identified in the brain tissue and placentas of children with microcephaly who died after birth, as well as in tissues from miscarried babies (Oliveira Melo *et al.*, 2016).

On March 30, 2016, the Brazilian Health Surveillance Agency (ANVISA) issued RDC-72 (Collegiate Board Resolution-72) (ANVISA 2016), the equivalent to a Resolution by the CDC, imposing mandatory IgM testing for ZKV prior to procedures involving germ cells and tissues, which includes ART clinics. Women with positive or undetermined test results are required to be retested within 30 days or to have a RT-PCR test performed at any time. Men with positive or undetermined test results are required to undergo both RT-PCR and semen testing at any time. Gametes can only be collected in ART procedures when test results are negative. This paper aims to discuss the RDC-72 from the standpoint of evidence and cost-effectiveness of the measures taken within the first two months of mandatory testing.

## MATERIAL AND METHODS

Fertipraxis is a private assisted reproduction clinic in Rio de Janeiro, the second largest city in Brazil and the host city of the Olympic Games held in August of 2016. An Excel spreadsheet containing the procedures scheduled to take place in April (at the time when the RDC-72 was issued), May, and June was analyzed, which included IVF and ICSI cycles, frozen embryo transfers, oocyte and semen cryopreservation, and intrauterine insemination (IUI) procedures.

The staff was gathered and the tasks divided among them. Operational team members were asked to list the laboratories in the city which were able to perform the tests and find out about their delivery times and costs, and the possibility of the tests being refunded by health insurance; nursing and medical assistants were asked to update patient data (continuous check), contact patients explaining the Resolution, decide what to do with ongoing cases, and find out the deadlines concerning the implementation of the new rules; laboratory personnel were asked to search for more information on the web, talk to the staff from other clinics, and collect information from the guidelines being produced. The staff met on a weekly basis to discuss their findings.

A SWOT (Strengths, Weaknesses, Opportunities, and Threats) analysis considering the positive and negative impacts of the new regulation upon the practices of a reproduction center was performed.

## RESULTS

Since the Since the first news on ZKV infection were published in late 2015, the patients seen in our clinic have been carefully probed for the possibility of infection and offered information about prevention by the medical and nursing staff during consultation.

An assistant physician called or emailed the patients needing treatment and invited them to undergo testing for ZKV infection.

In two months, 68 cases were tested and another 32 individuals had their final test results delivered, adding up to a total of 152 tests performed in three laboratories (Laboratorio Richet, www.richet.com.br; Laboratorios Lamina, Bronstein e Sergio Franco, DASA Group, www.dasa.com.br; and Labs a+, Fleury Group, www.fleury.com.br) chosen by the patients. Test results were handed in within three to 14 business days.

The following tests were carried out: (a) indirect immunofluorescence assay, IgM, Arbovirus Fever Mosaic; reference values: positive (>1/10), negative (≤1/10) or undetermined; 96.8% sensitivity and 96.6% specificity (Laboratories Richet and Fleury Group). (b) Serum indirect immunofluorescence assay, IgM, ZIKV; reference values: negative (<0.08), undetermined (0.08-1.09), and positive >1.09; weak cross-reactions possible with Nile fever, Dengue, and Yellow fever (DASA Group Labs). (c) Serum RT-PCR for ZKV, Biogene ZKV (all of them): reference value is not detected (<30 copies/mL), sensibility and specificity of 99.9% and urine RT-PCR (DASA Group Labs).

The estimated costs summarized in Table 2 were converted into American Dollars. Most health insurance plans do not process refunds for these tests, despite the existence of federal regulation granting them a mandatory status. Differences in test prices were found at the end of the first month when they were assessed for the second time. The third group also included tests for non-pregnant women.

**Table 2:** Testing prices, April and May of 2016.

	Lab1	Lab2	Lab3	Lab4	Lab5
Available on April 5, 2016	yes	yes	yes	yes	only in pregnant women
Possible health plan	Upon request	Upon request	Upon request	Upon request	
Detection Method PCR-RT	USD 113	USD 105	USD 102	USD 448	
	3 working days	14 working days	17 working days	10 working days	
IGM/IGG Each single detection	USD 61	USD 224	USD 93	USD 105	
	8 working days	10 working days	14 working days	4 working days	
PCR-RT	May 15, 2016				
	Same condition	USD 280	USD 257	USD 280	USD 154
	Same condition	6 working days	6 working days	4 working days	4 working days
IGM/IG Individual test price	Same condition	USD 61	USD 53	USD 60	USD 107
	Same condition	15 working days	15 working days	12 working days	20 days

Lab 1 - Richet; Labs 2-3 and 4- DASA Group; Lab 5 Fleury Group.

The Brazilian Health Surveillance Agency (ANVISA) was contacted by other assisted reproduction centers and asked how long negative test results were valid for. The answer, posted in the Embryo Mail Network, read as follows: "wait for a new statement; in the meantime, testing is still mandatory." In the first half of April, fifteen patients (of the 37 procedures performed during the month) were urged to take the tests, since they were already in the middle of their cycles when the Clinic received the notification from ANVISA. This resulted in significant stress to the patients.

By the end of the second month 152 tests had been performed and five cases were reported:

1. LNFM, 32, tested positive for ZKV in an emergency unit prior to starting an ART cycle, with symptoms observed in early February of 2016. The patient had a negative RT-PCR test 61 days after the first test, just before starting the stimulation protocol. She had an IVF/ICSI cycle resulting in clinical pregnancy followed by a miscarriage on May 23. She has one frozen blastocyst. On May 28, the OBGYN carried out a manual vacuum aspiration to retrieve specimens sent for testing for ZIKV. The patient lives in a town 298 km (186 mi.) away from Rio.
2. JMBF, 42, was suspected for ZKV infection on March 20 with unspecific symptoms. The couple had their cycle postponed. On May 18, the couple was tested for IgM. She was positive, although asymptomatic, and was tested for ZKV with serum and urine RT-PCR (DASA Group) and Dengue (immunofluorescence, IgG and IgM). Her test results came negative on May 24; the husband has not been tested yet.
3. RNBP, 35, was IgM positive on April 30, 2016 (Lab Richet) prior to an IVF cycle. Her husband tested negative. No symptoms were reported; the patient denied having episodes of fever or infection by dengue or chikungunya the previous year. Their cycle was postponed. RT-PCR was negative on May 24 (Lab Richet). The couple will start a cycle in June.
4. EDT, 34, was suspected for ZKV infection due to fever and one-day diarrhea on May 8, 2016 at an emergency unit. He was asked to test with RT-PCR on May 12. The cycle was postponed. The couple tested negative in the RT-PCR test (Lab Richet) on May 25, 2016. They will start a cycle in June.
5. PSS, 32, tested negative for IgM for an IVF/ICSI cycle (Lab Richet). Although informed, the husband postponed his tests and the lab could not issue the results on time for the retrieval procedure. The staff decided, as stated in the informed consent term, to retrieve and freeze oocytes only. On April 30 his results came negative and a cycle with frozen oocytes was started on May 16.

Direct costs involved in testing: mean price in April was USD 92.50 and USD 225 for IgM and PCR until mid May, when the prices dropped to USD 57 and USD 196.50 (Table 2). If a mean price per person of USD 100 were assumed, this means at least USD 15,200 of extra payment (USD 1.00 = BRL 3.50), or about USD 200 per couple. The woman tested with urine and serum RT-PCR at Laboratory 4 paid USD 448 for the tests. Table 3 shows the SWOT analysis.

**Table 3:** SWOT table Zika virus mandatory detection and ART in Brazil: Clinic and patients.

**Strenghtness**		**Weaknesses**
The team	Motivation Response capacity	Frustration Knowledge still limited
The patients	Sense of security on staff Welfare and careHigher costs Treatment motivation	False sense of post-testing safety
**Oportunities**		**Threats**
The team	Better understanding of the work Large multidisciplinary team activity Extra work	Results deadlines Postponed cycles
The patients	Prevention care in pregnancy	Anxiety of non-pregnancy Zika and microcephaly

On May 31, ANVISA promulgated a complimentary document, Technical Note no. 008/2016/GSTCO/GGMED/ DIARE/ANVISA, which provided guidance, among other things, on proven testing techniques or ZKV infection. However, we could not access the website where the information was supposedly posted.

## DISCUSSION

The exact incubation period for ZKV infection is unknown, but it probably lasts from a few days to a week. However, most people infected with the Zika virus will not know they have the disease, as in approximately 80% of the cases symptoms are not present (Portal Saude, 2016). The most common symptoms are fever, rash, joint pain, and conjunctivitis (red eyes), but patients may also have muscle pain and headaches.

On May 21, the Ministry of Health reported a total of 7,623 cases suspected for microcephaly in Brazil since the beginning of the investigation in October of 2015; 3,257 cases are still being investigated. Another 2,932 cases were ruled out for presenting normal test results or for having microcephaly and/or malformations for other reasons or for not meeting the case definition criteria. The 1,434 confirmed cases in Brazil occurred in 517 municipalities located in 25 Brazilian States. Two hundred and eight met the laboratory criteria for ZKV infection. Two hundred and eighty-five deaths are being investigated for possible connections with microcephaly and/or central nervous system disorders occurred after birth or during pregnancy (miscarriages and stillbirths). In 60 of them, microcephaly and/or central nervous system disorders were the confirmed cause of death. Another 187 are still under investigation and 38 cases were ruled out.

With so many unanswered questions, Health Authorities may end up having to be overly rigorous when issuing regulation. However, on May 28, the Portal da Saúde (Health Gateway) of the city of Rio de Janeiro had no notified cases of ZKV infection (Vigilância Epidemiológica - SMS, 2016). The only finding was the year-to-date 17,339 notifications of cases of dengue - in a population of 6,320,446 - versus the 10,000 cases reported last year.

Should asymptomatic patients be tested before undergoing ART procedures? Interesting reflections may be derived from the first two months of mandatory testing (Table 3) and, possibly, shed some light on this important question. The incidence of ZKV infection among asymptomatic patients was very low, and most clinics in Brazil are simply unable to comply with the testing requirements because of the continental proportions of the country and the lack of standardization in testing protocols. The Public Health Care System and private Health Insurance Companies do not process refunds for these tests, and they significantly increase the cost of treatment to patients. Interestingly, the cost of testing is equivalent to approximately to 1.2 day of treatment with controlled hyperstimulation drugs.

Diagnostic tests for ZKV infection are based on molecular and serologic methods (Interim Guidance, MMWR, 2016). Although RT-PCR is the preferred test today (as it can be performed rapidly and is highly specific), ZKV is unlikely to be detected in serum after the first week of disease and in urine for more than two weeks after the onset of symptoms. It has been indicated that further investigation is needed to determine the sensitivity and usefulness of these tests ≥14 days after onset of symptoms. While IgM tests might yield positive results from 5-7 days after infection, viremia has been described to occur for no longer than 11 days. Indeed, in the 152 tests performed, two were positive for IgM (1.3%) and both patients denied having symptoms or a history of dengue. The tests repeated after 25 and 60 days, as required by ANVISA, were negative (a female patient had negative serum and urine RT-PCR tests; a male patient suspected for ZKV infection had a negative RT-PCR test after 13 days).

Has there ever been a threat? Five couples had their cycles delayed for two months, one of which with a 42-year-old woman. And what can one say about asymptomatic couples trying to get pregnant without resorting to ART procedures? Should they be tested at all? If so, why? The guidelines in the MMWR indicate that routine testing is not recommended for individuals without clinical disease, once test results in this population tend to be less reliable (MMWR, 2016). Additionally, the considerable cross-reactivity of present flavivirus antibodies further hampers the interpretation of serologic test results.

None of the male patients in our series tested positive. It may be said that diagnosis based solely on clinical grounds added by public fear may overestimate the number of cases of disease. Even in the presence of symptoms and positive test results, the importance of testing is questionable. Musso *et al.* (2015), during the outbreak in French Polynesia, identified replicative viral particles, as well as viral RNA, often in high copy numbers up to 62 days after the onset of symptoms. As for testing, it is not clear whether a negative serum test result might rule out the presence of virus in semen; positive or negative semen test results might not suffice to support advice on conception. This is why the CDC guidelines recommend 120 days of observation from the detection of symptoms (Oduyebo *et al*., 2016).

Another interesting point is that mandatory testing for assisted reproduction has not been extended to blood banks. The site of the Brazilian Association of Hematology, Hematology and Cell Therapy (ABHH, 2016) shows a technical note advising blood banks on how to reduce the potential risk of virus transmission by blood transfusion. It cites the experience in French Polynesia (Musso *et al*., 2014) between 2013 and 2014, when 2.8% of voluntary blood donors tested positive for viral RNA, confirmed by nucleic acid amplification tests (NAATs) in 1,505 asymptomatic donors followed by standard care and repeats as provided to individuals with symptoms.

Quite a few questions remain unanswered, such as whether persons infected once are protected from future infections. The complexity around the decision of getting pregnant has only increased, specifically in countries affected by the disease. However, decisions are personal and clarification must be provided to the women and men willing to get pregnant in times of active ZKV transmission. The age of the woman in the decision to delay pregnancy is certainly a point of vital importance. Planning pregnancy is, more than ever, a must. Clinics must be prepared to counsel and advise patients on procedures such as freezing oocytes and/or embryos to have them transferred in months when milder temperatures and dryer days reduce the probability of mosquito bites.

## CONCLUSION

The team worked efficiently and responded promptly to the increased testing demand, but the tests failed to provide patients with more assurances over their safety and added to the cost of treatment. The time has come for the requirement to test patients prior to ART procedures to be reviewed along with the observation times applied to patient counseling over symptoms and cautionary measures required during pregnancy.
